# Notes and News

**Published:** 1887-11-05

**Authors:** 


### Notes and News.

Collected and Communicated.

The Earl of Derby laid the foundation-stone of the new Royal Infirmary at Liverpool on Saturday in the presence of a large assembly. The infirmary in course of demolition was erected in 1821, and was considered behind the age in regard to sanitary and other arrangements. The new one is to occupy an extended area, including the old site in Brownlow Street, and is estimated to cost £100,000, towards which £84,000 has been subscribed. In opening the proceedings on Saturday the Mayor expressed confidence that the money would be raised. The Bishop of Liverpool having offered up prayer, Lord Derby said that of all forms of charity hospital relief was the least likely to be abused. If they saw a man in rags it might not always be wise to supply him with a good suit of clothes until they knew how he came to be in that state of raggedness. But if they saw a man with a fever, or a broken arm or leg, there was no question how he came by the injury. They had him cured in the first place and inquired about the history afterwards. The common feelings of nature would not allow them to leave any human being to die of an accident or disease if medical or surgical aid could avail him. While present society lasted—and it was not likely to smash up yet—they would always have a vast multitude of men and women who could not pay for themselves the cost of careful medical attendance in a long sickness. They must be helped in some way, and hospital treatment was at once the least expensive and the most effective way. But there was something more to be said. It was no paradox to affirm that the greatest gainers by the existence of hospitals were to be found among those who never entered their doors. They were schools of medical science where a young man learning his business could see in a few months more cases of various kinds than he would be likely to see in the private practice of a lifetime; where he could profit by the teaching and experience of the leading men of his profession; where a high standard of professional feeling and honour was maintained; and where greed and quackery and all those practices by which low practitioners sometimes tried to push their way into notice were discountenanced and put down by the full force of an organised and unanimous opinion. Apart from the feeling of humanity to the individual it was impossible to overrate the public value of such services. An unhealthy people indicated a decay in the community, and the more they knew of former times the more surely they were aware that with the growth of civilisation life became on the average not only fuller but longer. These were the conclusions of medical and sanitary science, of all physicians and surgeons who gave a large proportion of their time and knowledge, which was their capital, to the unpaid relief of suffering among the poor. It was a peculiar pleasure to him to take part in the ceremony, for it was an ancestor of his who had laid the first stone of the original infirmary nearly 140 years ago; and it was his own godfather who 66 years ago laid the first stone of the building now being replaced.

The contract for furnishing throughout the new buildings of the Great Northern Central Hospital, in the Holloway Road, has been entrusted to Messrs. Jenks and Wood, of Berners Street, London.

We commence to-day a Student's Corner as suggested by one of the oldest and most experienced hospital managers. We shall always be glad to hear from students, and to supply them at any time, so far as possible, with any information or assistance that they many need.

Mr. J. Wilson Taylor writes: "Your remarks on the dangers of the dentist's chair in your issue of the 29th, apply with equal force to the 'barbers' chair. I know of two cases in my small experience where, through neglect in seeing that a clean cloth was laid on the head-rest of a barber's shaving chair, diseases of the head were contracted."

E. A. S. writes: "One feels it would be good if nurses' hours of work could be shortened; it is a pity 'to kill the goose with the golden egg.' That, I fear, is done in many places. From 6.30 a.m. to 9 p.m., with often not the allowed half-hour for meals, is surely too much. Doctors have little thought for nurses, and will ring the bell just as nurse has gone to dinner for a small matter that could well stand over. No wonder nurses have to be made to go out. No wonder they constantly say, 'Oh! I am so tired, may I not stay in my room?' In such small places as the one I superintend (fifty beds), it is with the small nursing staff quite impossible to allow nurses and probationers more than three hours on two days, and one hour on another day, during the week. It is all for the dear patients, supposing a few shillings be now and again given for drives. Do outsiders ever think how tired we nurses are; how we never get a breath of sweet morning air? Do they ever think what it must be to spend a lifetime amongst sick men and women; and, however much romance may be talked, patients are in real life far from the delightful creatures depicted in books? A real trustworthy nurse is worth good pay, and much consideration—two things sadly lacking in hospital work."

The third annual report of the Hospitals Association shows that the number of members and associates is steadily increasing, and that representatives of the most important hospitals, asylums, and nursing institutions are joining the association in considerable numbers. The scheme of Sir Andrew Clark, Bart., M.D., for increasing the funds of all the hospitals is now being brought under the notice of the clergy and ministers of all denominations and of the representative of other bodies, beginning with the metropolis. These proposals have excited a good deal of interest, and the following contributions towards the £1,000 required have already been given to enable the council to make them widely known and as helpful as possible: Messrs. Rothschild, £25; Mr. A. D. Clarke, £26 5*s.*; the Fishmongers' Company, £26 5*s.*; the Drapers' Company, £21; the Mercers' Company, £21; the Clothworkers' Company, £10 10*s.*; Messrs. Messel and Co., £10 10*s.* Sir Andrew Clark has himself contributed £100.

Miss C. F. Gordon Cumming, the well-known traveller, tells in her last published volume of an attempt to teach the blind in China to read, which has been successfully made by Mr. E. A. Murray, the agent of the National Bible Society in Pekin. His system consists of combinations, embosssed dots, and is strictly phonetic, so that the 4,000 characters used in Chinese typography are reduced to a comparatively small number of dots, representing certain sounds. Mr. Murray's first pupil, a street beggar, learned to read in six weeks, and it seems that blind pupils taught on Mr. Murray's plan learn more easily than those who see, owing to the greater simplicity of the method. This is a strong plea for some alteration in the present fashion of writing the Chinese language, in which the large number of symbols in use has hitherto been the stumblingblock in the path of the foreigner. Now that China is becoming opened up to the influences of Western civilisation there is a chance of some alteration in a language which, however voluminous, is lacking in phrases to express many of the needs of modern life and the means that exist to supply them.

Dr. William Murray, of Newcastle, has been alarming the inhabitants of that busy town by a lecture on what he calls "the evil effects of regular habits," which has utterly upset their previous ideas of wisdom and folly. It is exceptionally difficult to convince any man who leads a steady, industrious life, going to his shop or his office, and returning home, day after day, and year after year, at the same hours, that his course of existence may be every whit as suicidal as that of his most dissipated and erratic fellow-citizen. The good apprentice of Hogarth's pictures has become the model for succeeding generations of youth who look forward to riches, honour, and happiness as the natural results of industry. So they are, if the industry be judiciously varied by healthful amusement, travel, society, and rest for mind and body; without these it is more apt to bring in its train premature breakdown in some one of its numerous forms, with years of helplessness and pain, or early death. There is no lesson that the men of this age require to have impressed on them more strenuously than this, of the necessity for leisure and variety of interests to preserve the balance of physical and mental health; and Dr. Murray may feel that the controversy his lecture has occasioned is a proof that it contained a timely warning, which will rouse at least a few who were sacrificing their lives "to the god of getting on," to save them by a little harmless irregularity of habit.

On the 18th of last month a boy who was playing on the stone coping that ran round a building slipped, and fell upon the iron spikes that protected the coping. One of these entered his lungs, causing an injury of which the child died a week later. At the inquest, the jury, when returning a verdict of Accidental Death, stated that they considered the spikes dangerous, and requested the Coroner to convey this expression of their opinion to the owner of the building, together with a request for the removal of them. The twelve "good men and true" probably forgot, in their regret at the young life being so sadly cut short, that the coping of a building is not a public playground, and that the spikes were probably placed there to protect it from the invasion of such adventurous boys.

A death from hydrophobia which lately took place in Yorkshire deserves notice from the apparently trivial accident that caused it. The owner of a dog permitted his favourite to lick his hand when there was a slight abrasion upon it. Shortly afterwards the dog showed symptoms of rabies, and was killed; subsequently, the master developed hydrophobia, which terminated fatally. Such a case as this makes us speculate whether the master would have suffered from the disease if he had not known of his dog's madness. In many cases what is called hydrophobia is simply fear. Extreme terror can kill as well as any other accident, and the universal interest aroused by M. Pasteur's experiments tends to produce imaginary hydrophobia, a disease which is only one of the forms of hysteria, but, none the less, may result fatally with persons of morbid and fearful temperament.

A punishment in vogue among English schoolboys has been incorporated into the discipline of the French army. A newly-enrolled dragoon at Valenciennes having failed to get through his riding lesson creditably, was, by order of the sergeant, tossed in a sheet. He was a stout and heavy man, and after having been tossed, he fell with such force that he tore the sheet and came to the ground, sustaining severe injuries. He was at once conveyed to the hospital, where he still remains in a critical condition. This is not the only experiment in tossing that has been made. Three cuirassiers were lately subjected to the same punishment, one of whom has since died from injuries then received. The shoulder of another was dislocated, and the third had his leg broken. No doubt "tossing" is a very effective way of reducing the strength of standing armies, but it is not one likely to meet with general approval. There is folly as well as brutality in allowing punishment to degenerate into reckless cruelty.

Sir Andrew Clark's scheme for increasing the voluntary support of the hospitals is one which would have been entirely after the late Dr. Wakley's heart. It is beginning to be realised how great a loss Dr. Wakley's death is likely to prove to the metropolitan hospitals, especially when it is remembered that, as editor of the *Lancet*, he never lost an opportunity of advocating, not once only, but from week to week, the necessities of the hospitals, and the duty of affording their managers adequate pecuniary support. He it was who wrote and wrote and wrote again until London aroused itself to the importance of establishing the Metropolitan Hospital Sunday Fund. This movement in connection with The Hospitals Association, by widening the area of interest in the work of hospitals, must tend to produce additional funds from voluntary sources. All who know the needs and the value of hospitals will welcome and support the scheme, whether as private individuals or as members of the press. We can only hope that our contemporaries will examine this scheme closely and then we have no doubt they will give it the benefit of their influential advocacy.

A communication we have received from a brother editor reminds us of an article which appeared in vol. i., page 122, in which an account is given of a newspaper called the *Looking Glass*. He sends us a slip which has been sent him, purporting to be an article on "Pensions and Insurance Funds for Nurses," from the *Charity Record*. The article deliberately argues that "Mr. Burdett desires to look in all directions for eleemosynary aid." The writer's aim is evidently to discredit the effort which nurses and the officials of hospitals and other benevolent institutions are making to provide for the day of sickness and old age by united action on a non-eleemosynary basis. The statements in the article in question are either false, or misleading. The print in which they appears has, however, only to be seen to be recognised as belonging to a type of journals now happily almost extinct. It has tried more than once to discredit, and so to diminish the contributions to the hospitals through the Hospital Sunday Fund. Our *confrère* who sends us the slip states that "some one has taken the trouble to circulate it, sending it to a great many newspapers." No doubt the officers and nurses of public institutions will appreciate the action of the print in question, and we hope those editors who have received these slips will take the trouble to obtain a copy of the *Charity Record*, and so judge for themselves what weight can justly be attached to its utterances.

The circulation of Sir Andrew Clark's scheme is bringing to light some little-known agencies which exist to raise funds for hospitals. The eleventh annual report of one such, the Mutual Friendly Aid Society, has been sent us by the President, Mr. T. Parrant, of Bow, N. This Society raised £318 for the hospitals last year, the expenditure being less than eight per cent. of that sum, and £294 was given to the London, the City of London, Victoria Park, the German, and the Hackney Road Children's Hospitals, the Adelaide Dispensary, and the Truss and Surgical Aid Societies, which institutions it was founded to assist with funds. The Society has during the eleven years of its existence collected £1,860, and has nominated some 160 governors to the institutions benefited. It has set every congregation and parish throughout the kingdom an example which they might usefully follow by adopting the scheme Sir Andrew Clark has set forth in another column of the present issue. We would suggest that future reports of the Mutual Friendly Aid Society should contain an account of the methods adopted for raising money, together with an outline of the Society's origin and progress.

Our contemporary, the *Echo*, is so ably conducted that we are sorry to see it taking a doubtful departure. This consists in making quotations from our columns, an honour we should better appreciate if the writer of the notes in question did not omit to mention the source from which he gets his inspiration and facts. It is true he vaguely alludes to "a medical paper," but as there are several journals which come under this category, we hope he may be moved in the future to give the title of the particular paper from which he is quoting at the moment. We say this in all courtesy and frankness, and because we know our hospitals are indebted to the *Echo* for much kindly help on many occasions.

How few people there must be who have not heard of, and who do not frequently use, *Whitaker's Almanack!* We have had more than one experience of Mr Whitaker's generosity and public spirit, and it is therefore a pleasure to record that the forthcoming edition of his almanac will contain an account of all the hospitals and kindred charities in such a form as to ensure them more liberal public support in the future. We believe the original proposal came from Mr. F. C. Carr-Gomm, the energetic chairman of the London Hospital, who has kindly undertaken to collect the additional information, and to arrange it in a form suitable for publication in the almanac. Hospital managers will be duly grateful for this kindly aid at a critical time. Indeed, Mr. Whitaker's action may be held to prove the increasing interest which the public are taking in the development and prosperity of hospitals of all kinds.

Mr. Saxon Snell and other early opponents of the circular form of hospital wards used to argue, in effect, that no responsible hospital committee could venture to accept plans for buildings on the circular principle on account of the increased expenditure (*a*) on the prime cost for building due to the expensive character of circular work; and (*b*) on the after administration of these wards. The Miller Memorial Hospital at Greenwich was the first circular hospital erected, and it proved that the original cost of the buildings was smaller than that of many rectangular hospitals. Indeed, it did not exceed £170 per bed. The new circular hospital at Hastings, including the expensive flat roofs, the large balconies, the disconnecting lobbies to the wards, the isolation wards, the out-patients' department which, by the bye, is the fly in the ointment, and other special features, will not cost more than £250 per bed. Of this sum we have ascertained that an expenditure equal to £15 per bed has been specially incurred by the committee with the view of extending the decorative features of the building, owing to the commanding position which it occupies in the centre of this popular watering-place. As to the cost of administering hospitals built with circular wards, Mr. Burdett shows on p. 101 that the saving in wear and tear and physical exertion to the nurses and other resident members of the staff cannot fail to have an appreciable effect on their general health, and consequently on the efficiency of their work.

The fifteenth annual report of St. Mary's Cottage Hospital, Northam, Southampton, testifies to much good work thoroughly well done. Mrs. T. Black founded this little hospital in 1872, for the purpose of treating sore legs of long standing. An account of it will be found in vol. i., page 316. It is, we believe, the only hospital of its kind. Nearly 500 patients have reaped the benefits of the hospital during the year, of whom nearly 400 were cured, and upwards of 100 still remain under treatment. The cures seem to be permanent, as only two relapses occurred during the year. About 6,000 dressings have been applied at the hospital, and nearly 1,400 "have been sent by post or carrier to patients who from great distances are able to attend only occasionally." In 1886, the ordinary receipts amounted to £269, and the ordinary expenditure to £280. Patients have been received from various parts of the metropolis, from as far north as Nottingham, and as far south as the Isle of Wight. This is practical testimony to the utility of Mrs. Black's excellent institution.

We regret that we cannot publish Mrs. Buchanan's letter on the loss of her daughter (see pp. 17 and 38, vol. iii., and p. 322 vol. ii.), not only owing to its extreme length, but also because it contains a charge against the Committee of Visitors of the Warwick Asylum, which we can hardly be expected to print in our columns. Moreover, the whole question, as our correspondent will see on reflection, is a legal and not a medical one, hence it should be placed in the hands of her legal advisers. We may, however, answer the question put to us about satisfying any three of the Visiting Justices, by simply giving the following list of the Committee: The Lord Leigh, the Lord Dormer, T. C. S. Kynnersley, Esq., J. T. Arkwright, Esq., Thomas Lloyd, Esq., D. Galton, Esq., J. C. Bucknill, Esq., M.D., F. W. Arkwright, Esq., J. Machen, Esq., F. J. M. Mason, Esq., J. B. Dugdale, Esq., J. Y. Robins, Esq., the Hon. Charles Adderley, S. S. Lloyd, Esq., and M. H. Lakin, Esq. It is hardly possible to believe that the above could be induced by any wrong motive to detain the patient; and we repeat that it is only necessary to obtain the signatures of *any three of them* to ensure the discharge of the patient. We notice especially that the list contains the name of Dr. J. C. Bucknill, formerly Lord Chancellor's Visitor in Lunacy, and a man not likely to be deceived. In reply to Mrs. Buchanan's second letter, we have to point out: (1) The Committee of Visitors have power to make any rules for the management of their asylum, provided such rules do not conflict with the Lunacy Acts. If our correspondent was priviliged to visit her daughter in the wards on one occasion, it is no reason why she should always have it; besides, is she sure that the privilege was not in some way or other abused? (2) Clearly the friends of patients cannot expect refreshments. There are probably 600 patients in the Asylum, and to supply food to the friends of all these would entail considerable expense to the ratepayers, nor is there any section of the Lunacy Acts which permits such expenditure.

